# Molecular evidence for stimulation of methane oxidation in Amazonian floodplains by ammonia-oxidizing communities

**DOI:** 10.3389/fmicb.2022.913453

**Published:** 2022-08-01

**Authors:** Gabriel G. T. N. Monteiro, Dayane J. Barros, Gabriele V. M. Gabriel, Andressa M. Venturini, Tomás G. R. Veloso, Gisele H. Vazquez, Luciana C. Oliveira, Vania Neu, Paul L. E. Bodelier, Cleber Fernando M. Mansano, Siu M. Tsai, Acacio A. Navarrete

**Affiliations:** ^1^Federal Rural University of Amazonia (UFRA), Belém, Brazil; ^2^Graduate Program in Biodiversity and Biotechnology (BIONORTE), Federal University of Tocantins (UFT), Palmas, Brazil; ^3^Graduate Program in Biotechnology and Environmental Monitoring, Federal University of São Carlos (UFSCar), Sorocaba, Brazil; ^4^Cell and Molecular Biology Laboratory, Center for Nuclear Energy in Agriculture, University of São Paulo (USP), Piracicaba, Brazil; ^5^Princeton Institute for International and Regional Studies, Princeton University, Princeton, NJ, United States; ^6^Graduate Program in Agricultural Microbiology, Federal University of Viçosa, Viçosa, Brazil; ^7^Graduate Program in Environmental Sciences, University Brazil, Fernandópolis, Brazil; ^8^Department of Physics, Chemistry, and Mathematics, Federal University of São Carlos (UFSCar), Sorocaba, Brazil; ^9^Department of Microbial Ecology, Netherlands Institute of Ecology (NIOO-KNAW), Wageningen, Netherlands

**Keywords:** ammonia oxidation, methanotrophs, Thaumarchaeota, 16S rRNA sequencing, tropical floodplains

## Abstract

Ammonia oxidation is the rate-limiting first step of nitrification and a key process in the nitrogen cycle that results in the formation of nitrite (NO_2_^–^), which can be further oxidized to nitrate (NO_3_^–^). In the Amazonian floodplains, soils are subjected to extended seasons of flooding during the rainy season, in which they can become anoxic and produce a significant amount of methane (CH_4_). Various microorganisms in this anoxic environment can couple the reduction of different ions, such as NO_2_^–^ and NO_3_^–^, with the oxidation of CH_4_ for energy production and effectively link the carbon and nitrogen cycle. Here, we addressed the composition of ammonium (NH_4_^+^) and NO_3_^–^—and NO_2_^–^—dependent CH_4_-oxidizing microbial communities in an Amazonian floodplain. In addition, we analyzed the influence of environmental and geochemical factors on these microbial communities. Soil samples were collected from different layers of forest and agroforest land-use systems during the flood and non-flood seasons in the floodplain of the Tocantins River, and next-generation sequencing of archaeal and bacterial 16S rRNA amplicons was performed, coupled with chemical characterization of the soils. We found that ammonia-oxidizing archaea (AOA) were more abundant than ammonia-oxidizing bacteria (AOB) during both flood and non-flood seasons. Nitrogen-dependent anaerobic methane oxidizers (N-DAMO) from both the archaeal and bacterial domains were also found in both seasons, with higher abundance in the flood season. The different seasons, land uses, and depths analyzed had a significant influence on the soil chemical factors and also affected the abundance and composition of AOA, AOB, and N-DAMO. During the flood season, there was a significant correlation between ammonia oxidizers and N-DAMO, indicating the possible role of these oxidizers in providing oxidized nitrogen species for methanotrophy under anaerobic conditions, which is essential for nitrogen removal in these soils.

## Introduction

Methane (CH_4_) is a greenhouse gas with approximately 34 times greater global warming potential than carbon dioxide (CO_2_) over 100 years, and since 1750, atmospheric levels of CH_4_ have increased by 157% (Myhre et al., 2013; World Meteorological Organization [WMO], 2021). Some of the largest natural sources of CH_4_ are wetlands, which account for 175–217 Tg CH_4_ yr^–1^ (Ciais et al., 2009; Kirschke et al., 2013), which is approximately 30% of the total emissions (Bloom et al., 2010, 2012). Tropical and subtropical wetlands are important sources of CH_4_ because of their elevated net primary productivity and high temperatures, producing 50–60% of all wetland CH_4_ emissions (Walter et al., 2001; Bloom et al., 2010, 2012).

Methane is the net result of the production and consumption of methane by methanogenic and methanotrophic microorganisms, respectively. This balance can be shifted by the flood pulse that changes the soil water saturation and favors methanogenic or methanotrophic activity depending on climatic conditions, land use, and anthropogenic activities (Steudler et al., 1996; Dalal and Allen, 2008; Meyer et al., 2017). When the soil is flooded, anoxic conditions induce CH_4_ production by methanogenic microorganisms (Agostinetto et al., 2002). However, under aerobic and anaerobic conditions, CH_4_ can be oxidized to CO_2_ by methanotrophic microorganisms, which act as the primary biological sinks of this greenhouse gas (Knittel and Boetius, 2009).

Anaerobic oxidation of methane is an important sink of this atmospheric gas (Conrad, 2009), as it plays an essential role in global warming mitigation. AOM was first found to be coupled with sulfate reduction and mediated by anaerobic methanotrophic archaea (ANME) and sulfate-reducing bacteria (SRB) (Boetius et al., 2000; Cui et al., 2015). Currently, AOM has been observed to be coupled with different terminal electron acceptors, such as nitrate (NO_3_^–^) (Haroon et al., 2013), nitrite (NO_2_^–^) (Ettwig et al., 2010), and other metals, including iron and manganese (Beal et al., 2009; Gabriel et al., 2020), which effectively link nitrogen and/or metal cycles to CH_4_ consumption.

Nitrate/nitrite-dependent anaerobic methane oxidation (N-DAMO) is mediated by the members of the bacterial and archaeal domains. Nitrite-dependent AOM is mediated by *Candidatus* Methylomirabilis oxyfera, a member of the NC10 bacterial phylum (Raghoebarsing et al., 2006; Ettwig et al., 2009), while nitrate-dependent AOM is catalyzed by anaerobic archaea belonging to the ANME-2d clade, *Candidatus* Methanoperedens nitroreducens (Haroon et al., 2013). The latter uses a reverse methanogenesis pathway for CH_4_ oxidation, with methyl-coenzyme M (methyl-CoM) reductase as the key enzyme, followed by NO_3_^–^ reduction to NO_2_^–^ (Haroon et al., 2013; Arshad et al., 2015; Ettwig et al., 2016).

A study in the Amazonian floodplains revealed a disproportionate role of the NC10 phylum in CH_4_ mitigation across soil layers, forest, and agricultural sites, with seasonal flooding in the different water types of the floodplains and the presence of *Candidatus* M. nitroreducens in the clear water floodplain of the Tocantins River (Bento et al., 2021). The nitrate-reducing archaeon *Candidatus* M. nitroreducens can not only reduce NO_3_^–^ to NO_2_^–^ but can also perform dissimilatory nitrate reduction to ammonium (DNRA) (Ettwig et al., 2016). It also shows syntrophic relationships with anammox (anaerobic ammonium oxidation) bacteria and *Candidatus* M. oxyfera depending on the substrate availability (Haroon et al., 2013).

In this study, we revealed the composition of active ammonia-oxidizing and nitrogen-dependent anaerobic methane-oxidizing communities using high-throughput sequencing of 16S rRNA transcript amplicons recovered from flooded and non-flooded forest and agroforest soils in an Amazonian clear water river floodplain. We hypothesized that (*i*) soil chemical factors, depth, land use, and seasonality modulate the active AOA and AOB communities and the occurrence of nitrogen-dependent anaerobic methane oxidizers in these soils, and (*ii*) increases in AOA and AOB abundance are coupled to an increase in abundance of nitrogen-dependent anaerobic methane oxidizers in flooded and non-flooded soils.

## Materials and methods

### Site description and soil sampling

Sampling sites were identified as primary forests (*i.e.*, one forest site with no visible indications of human activities or significant disturbance of ecological processes) or traditional farming systems (*i.e.*, one traditional cocoa-based agroforestry site) and characterized using: (1) Shuttle Radar Topography Mission (SRTM) satellite images at a resolution of 1 arc-sec (30 m), (2) Landsat 8 satellite data, (3) Google Earth images with Universal Transverse Mercator (UTM) projection, (4) SIRGAS referencing (UTM zone 22S to 20S), (5) field visits, and (6) QGIS v.2.18 “Las Palmas” geoprocessing tools. These sites are located in the lower Tocantins basin, municipality of Baião (2°40′51″ S, 49°39′05″ W) in the state of Pará, Brazil, in the hydrographic basin of the Tocantins River. This basin extends from 46° to 55°W and 2° to 18°S, with a total drainage area of 918 822 km^2^, and this represents 11% of the total Brazilian territory (Agência Nacional de Águas [ANA], 2015). Both sites are in an area that is seasonally flooded with clear water from the Tocantins River.

The study area is downstream of the Tucuruí Hydroelectric Power Plant reservoir, the largest Brazilian hydropower dam, with a flooded area of 2,850 km^2^ (Agência Nacional de Águas [ANA], 2009). Both sites are in an area seasonally flooded with clear water from the Tocantins River. After the construction of the Tucuruí dam, a reduction was recorded in the extension of the areas flooded by the river bed downstream, as a consequence of the regulation of the flow caused by the damming of the natural course of the river (Junk and Mello, 1987). The lower Tocantins River has a semi-diurnal tidal effect that alters river hydrodynamics (De Mérona et al., 2010). These tidal effects increase the water’s residence time and connection between the riparian zone, floodplains, and the main river channel. The topography of the basin is dominated by gently sloping upland plateaus, and the soils consist of Oxisols (24%), Ultissol (17%), Entisols (23%), and Entisol Plinthic (14%) (Agência Nacional de Águas [ANA], 2009).

The climate in this basin is tropical (hot and humid), with strong seasonality (Gomes et al., 2019). The annual average temperature is 26°C and precipitation is 2,533 mm (Agência Nacional de Águas [ANA], 2009). According to the Köppen climate classification, four distinct climate types are identified in the basin: Af in the northern region (tropical climate without dry season), Am in the northwest (with a moderate drought season between July and September), and Aw in the northwest, central-west, and east regions, which have an annual rainfall of approximately 1,700 mm, with a dry season between June and August. The last climate type is Cwa, humid subtropical with a dry winter and hot summer, which corresponds to the savanna area (Alvares et al., 2013).

In the primary forest and the adjacent traditional cocoa-based agroforest, soil samples were collected at three points that were a minimum of 14 m apart along a transect. Undeformed soil cores were taken from 0 to 30 cm topsoil layer in the dry (October 2017) and flood (May 2018) regimes of the floodplains. During the dry season, soil samples were collected in an aseptic cylindrical core (0–30 cm deep and 10 cm diameter) with a threaded partition at 15 cm (Supplementary Figure 1A). During the flood season, underwater soil cores (0–30 cm deep and 10 cm diameter) were collected using a universal hand core sediment sampler with a 0–15 cm adjustable core cutter (Aquatic Research Instruments, ID, United States) (Supplementary Figure 1B).

Soil cores collected in both the dry and flood regimes of the floodplains were sliced into two 15 cm layers (0–15 cm depth and 15–30 cm depth), totaling 24 soil samples (two soil layers × three sampling points × two land uses × two seasons). A subsample containing 3 g of soil was collected from each of the two 15 cm soil layers per point, immediately preserved in LifeGuard Soil Preservation Solution (Qiagen, Hilden, Germany), transported to the laboratory on ice, and stored at −80°C until further processing within 72 h after sampling. The remaining soil from each layer was used for the physicochemical analysis.

### Soil chemical factor measurement and analysis

Chemical measurements were performed on each of the 24 soil samples. Soil pH was measured using a soil suspension in 0.01 M CaCl_2_ (1:5, w/v). Soil moisture was determined using the gravimetric method described by Silva (2009), dissolved organic carbon (DOC) content using a Shimadzu TOC-5000A analyzer (Shimadzu, Columbia, MD, United States), and total carbon (C) and nitrogen (N) contents by dry combustion using a CHNS/O elemental analyzer (PerkinElmer, Waltham, MA, United States) from the soil samples. The analyses were performed using 5–7 mg of dry soil sieved through a 0.15-mm mesh. Sulfate was extracted and determined according to Cantarella and Prochnow (2001). Soil ammonium and nitrate were extracted using 2 M potassium chloride (KCl) and quantified by spectrophotometry as described by Krom (1980) and Norman et al. (1985), respectively.

### Soil RNA isolation, cDNA synthesis, and 16S rRNA gene amplicon sequencing

Total RNA was isolated from 2 g of soil (wet weight) of each of the 24 soil samples using the RNeasy PowerSoil Total RNA Kit (Qiagen) according to the manufacturer’s instructions. RNA concentration and purity were assessed spectrophotometrically (Nanodrop ND-1000, Nanodrop Technologies, Inc., Wilmington, DE, United States) to determine the absorbance at 230, 260, 280, and 320 nm. The concentration of isolated RNA was 100–200 ng μL^–1^. RNA extraction was performed in duplicate for each sample (two technical replicates) and stored at −80°C until further use.

Complementary DNA (cDNA) was synthesized from single-stranded RNA using a QuantiNova Reverse Transcription Kit (Qiagen) with integrated removal of genomic DNA contamination following the manufacturer’s instructions.

Amplicon libraries were prepared using 16S Metagenomic Sequencing Library Preparation,^1^ with cDNA as a template in the amplification reactions and specific primers for the archaeal and bacterial 16S ribosomal RNA (rRNA) genes. The primer pair SD-Arch-0349-aS-17 (5′-GYGCASCAGKCGMGAAW-3′)/SD-Arch-0519-aA-16 (5′-TACCGCGGCKGCTG-3′) (Klindworth et al., 2013) was used to obtain amplicons of approximately 185 bp from the V3 region of the archaeal 16S rRNA gene. A fragment of approximately 390 bp of the V4 region of the bacterial 16S rRNA gene was amplified using the primers 515F (5′-GTGYCAGCMGCCGCGGTAA-3′) (Parada et al., 2016) and 806R (5′-GGACTACNVGGGTWTCTAA-3′) (Apprill et al., 2015).

Each reaction contained 2.5 μL of 10 × reaction buffer, 1 μL of MgCl_2_ (50 mM), 1 μL of dNTP (10 mM), 1 μL of bovine serum albumin (BSA; 1 mg mL^–1^), 0.5 μL of each primer (10 μM), 10 ng of template cDNA, 0.5 μL of Platinum Taq DNA Polymerase (5 U μL^–1^) (Thermo Fisher Scientific, Waltham, MA, United States), and sterile water to obtain a final volume of 25 μL. The thermocycling conditions were as follows: initial denaturation for 3 min at 95°C, 30 cycles of 30 s at 95°C, 30 s at 58°C for archaea (60°C for bacteria), 30 s at 72°C, and a final extension for 10 min at 72°C. All primers contained the Illumina adapter sequences. PCR products were purified using Agencourt AMPure XP beads (Beckman Coulter, Inc., Brea, CA, United States) and resuspended in nuclease-free water.

Amplicons were quantified using the 1000 Assay of the Agilent 2100 Bioanalyzer System (Agilent Technologies, Santa Clara, CA, United States). Amplicons from the 24 samples were indexed in a PCR reaction with 20 cycles using the Nextera XT Index Kit (Illumina, San Diego, CA, United States) and KAPA HiFi HotStart ReadyMix (Roche, Pleasanton, CA, United States). The 24 multiplexed samples were pooled at equal volumes by library, which was performed separately for archaea and bacteria. The pooled libraries were purified using HighPrep™ PCR Magnetic Beads (MagBio Genomics, Gaithersburg, MD, United States) and subsequently assayed on an Agilent 2100 Bioanalyzer System (Agilent Technologies) to estimate library size. Libraries were quantified using the Qubit™ dsDNA HS (High Sensitivity) Assay Kit on a Qubit 2.0 fluorometer (Life Technologies, Carlsbad, CA, United States) and KAPA SYBR^®^ FAST qPCR Master Mix and Illumina standards and primer premix (KAPA Biosystems, Wilmington, MA, United States), according to the Illumina suggested protocol.

The resulting 16S rRNA amplicon libraries were denatured with 10 μL of NaOH, diluted to 8 pM with the Illumina HT1 buffer, and spiked with 1 % PhiX (Illumina). Equal concentrations of libraries were loaded independently for the 16S rRNA amplicons of archaea and bacteria using the MiSeq Reagent Kit v3 (Illumina). The equipment used for 16S rRNA amplicon sequencing was a MiSeq Personal Sequencing System (Illumina) operated in a rapid run mode to generate 2 × 300 bp paired-end reads.

### 16S rRNA amplicon sequence data preprocessing and taxonomic determination

All 16S rRNA gene sequence reads were processed and analyzed using QIIME v.1.9.1 (Quantitative Insights into Microbial Ecology) software (Caporaso et al., 2010a). Briefly, fastq files with forward and reverse reads were merged using the UPARSE algorithm (Edgar, 2013). Sequences that did not merge with this algorithm were merged using VSEARCH v.2.10.4, with 2 bp as the minimum length for overlap. The merged reads were further preprocessed by (*i*) quality control of the consensus sequences using a *Phred* quality score of 20 for each base call (Ewing and Green, 1998), (*ii*) removal of artificial sequences, such as primers and adapters, (*iii*) disposal of short length reads with less than 100 bp (archaea) and 200 bp (bacteria), and (*iv*) removal of ambiguous sequences. USEARCH (Edgar, 2010) was then employed to remove chimeras from the preprocessed reads. After chimera removal, the preprocessed reads were aligned using PyNAST (Caporaso et al., 2010b) with SILVA Release 132^2^ and sorted with >97% similarity into operational taxonomic units (OTUs) using a closed-reference OTU picking approach.

### 16S rRNA amplicon sequence data analysis

To reveal the active nitrogen-dependent anaerobic methane-oxidizing (N-DAMO) and ammonia-oxidizing microorganisms, the number of 16S amplicon sequences in each library was normalized using the OTU table (2,500 sequences for archaea and 20,000 sequences for bacteria) and the rarefaction method of QIIME v.1.9.1. The OTU tables obtained for the 16S rRNA gene sequences of archaea and bacteria were used separately to filter *Candidatus* Methanoperedens nitroreducens *and Candidatus* Methylomirabilis oxyfera clusters, as well as the archaeal and bacterial ammonia-oxidizing communities using the “filter_taxa_from_OTU_table.py” command of QIIME v.1.9.1 and the script described by Pylro et al. (2014). The relative abundances of active ammonia oxidizers and N-DAMO taxa were estimated by dividing the number of sequences belonging to each by the total number classified as ammonia oxidizers or N-DAMO per sample.

### Statistical analyses

Statistical analyses were conducted using PAST software (Hammer et al., 2001) and R studio 4.0.5 (RStudio Team, 2018). Statistical analysis and graphical visualization in the R studio were performed using “vegan” 2.5-7 (Oksanen et al., 2022), “ggpubr” 0.4.0 (Kassambara, 2020), and “ggplot2” 3.3.5 (Wickham and Chang, 2016). Tukey’s HSD *post-hoc* analysis was used to determine the significance of the differences between the non-flood and flood seasons of the floodplain, and between the forest (FOR) and agroforest (TFS) for each soil factor within each soil layer (0–15 cm and 15–30 cm). The similarity between samples regarding their soil chemical factors and ammonia-oxidizing and nitrogen-dependent anaerobic methane-oxidizing community composition was assessed using nonmetric multidimensional scaling (NMDS) and permutational multivariate analysis of variance (PERMANOVA), using Euclidean distance for the chemical attributes and Bray-Curtis distance for community composition. Further evaluation of the contribution of land use, soil depth, and seasonality to the total variation in ammonia-oxidizing and nitrogen-dependent anaerobic methane oxidizer communities was determined through variance partitioning analysis (Borcard et al., 2018). The significance of each chemical factor was assessed using PERMANOVA. Pearson’s correlation was used to confirm the relationship between ammonia-oxidizing and nitrogen-dependent anaerobic methane oxidizers.

## Results

### Soil chemical profile

**FIGURE 1:**
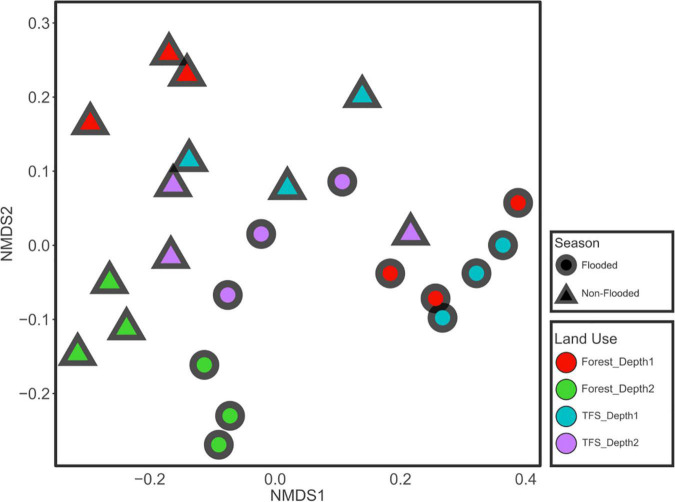
Clustering of soil chemical properties during different seasons (flood and non-flood) and different combinations of sites and depths. The plot is based on nonmetric multidimensional scaling (NMDS) using the Euclidean distance index.

Lower pH values (*p* < 0.05) were observed in the FOR site compared to the TFS site in both flood and non-flood seasons, whereas sulfate and the C:N ratio were significantly higher at the FOR site, primarily during the non-flood season. Other chemical factors varied across seasons, sites, and depths (Table 1). Nonmetric multidimensional scaling-based multivariate analysis indicated that the contrasting seasons (*R*^2^ = 0.30, *p* = 0.001), sites (*R*^2^ = 0.10, *p* = 0.001), and depths (*R*^2^ = 0.23, *p* = 0.001) had highly different chemical profiles. The interaction between depth and season and between depth and sites were also significant (*R*^2^ = 0.095 and 0.055; p = 0.004 and 0.021, respectively), although only a small portion of the variance was explained (Figure 1 and Table 2).

**TABLE 1:** Chemical attributes of the soil samples at each depth (0–15 and 15–30 cm), site [Forest (FOR) and Agroforest (TFS)], and season [Non-flood (NF) and Flood (F)] in the Tocantins River plain.

Chemical factors	Depth (cm)	Non-flood		Flood		NF vs. F*	
				
		FOR	TFS	FOR	TFS	FOR	TFS
pH	0–15	3.65b ± 0.01	3.97a ± 0.04	3.75b ± 0.01	4.08a ± 0.09	*	ns
	15–30	3.67b ± 0.06	3.80a ± 0.04	3.85a ± 0.09	4.05a ± 0.20	*	ns
Sulfate	0–15	0.51a ± 0.02	0.38b ± 0.04	0.35a ± 0.04	0.31a ± 0.04	*	ns
	15–30	0.43a ± 0.04	0.36b ± 0.02	0.40a ± 0.10	0.25a ± 0.04	ns	*
Ammonia	0–15	6.08a ± 2.85	13.0a ± 9.21	31.4a ± 17.96	24.35a ± 5.28	*	ns
	15–30	1.44a ± 0.52	8.98a ± 9.32	3.41a ± 0.50	8.22a ± 5.23	ns	*
Nitrate	0–15	9.34a ± 2.03	4.20b ± 1.52	0.25a ± 0.20	0.07a ± 0.06	*	*
	15–30	1.25a ± 0.59	1.93a ± 1.49	0.17b ± 0.26	1.41a ± 0.71	*	ns
Total carbon	0–15	2.05a ± 1.04	1.37a ± 0.14	1.49a ± 0.69	1.14a ± 0.85	ns	ns
	15–30	0.77a ± 0.36	1.01a ± 0.04	0.42a ± 0.03	0.73a ± 0.31	ns	ns
Total nitrogen	0–15	0.16a ± 0.09	0.13a ± 0.02	0.12a ± 0.05	0.10a ± 0.06	ns	ns
	15–30	0.05b ± 0.02	0.10a ± 0.005	0.03b ± 0.004	0.08a ± 0.02	ns	ns
C:N ratio	0–15	11.54a ± 0.25	10.14b ± 0.16	12.52a ± 0.23	10.17a ± 7.60	*	ns
	15–30	11.24a ± 0.30	9.24b ± 0.17	12.43a ± 0.71	9.10b ± 3.54	ns	ns
DOC	0–15	7.39a ± 1.52	5.60a ± 0.66	8.30a ± 1.87	3.87b ± 0.77	ns	*
	15–30	5.73a ± 0.76	5.38a ± 0.59	4.03a ± 0.49	4.30a ± 1.43	*	ns

NF, non-flood; F, flood; N, nitrogen;f DOC, dissolved organic carbon. pH, sulfate concentration (mg kg^–1^), ammonia (mg kg^–1^), nitrate (mg kg^–1^), total carbon (%), total nitrogen (%), carbon:nitrogen (C:N) ratio, dissolved organic carbon (DOC) (mg kg^–1^). Values with different letters are significantly different (*p* < 0.05) based on Tukey’s HSD test. Tukey’s test was performed with FOR vs. TFS within each flood regime.

*Tukey’s test was performed with NF vs. F samples within each land use site. Bold letters are significant (*p* < 0.05). The results represent the mean of three values ± standard deviation.

*Significantly different (*p* < 0.05).

ns = non-significantly different (*p* > 0.05).

**TABLE 2:** Permutational multivariate analysis of variance of the chemical properties and the microbial community profiles in the floodplain of the Tocantins River.

Data	Season			Site			Depth			Season × Depth			Site × Depth		
					
	R^2^	F	*P*-value	R^2^	F	*P*-value	R^2^	F	*P*-value	R^2^	F	*P*-value	R^2^	F	*P*-value
Chemical profile	0.305	25.941	**0.001**	0.099	8.467	**0.001**	0.226	19.221	**0.001**	0.095	8.098	**0.004**	0.055	4.673	**0.021**
Ammonia-oxidizing taxa	0.103	4.041	**0.001**	0.115	4.506	**0.001**	0.110	4.322	**0.001**	0.062	2.429	**0.005**	0.075	2.953	**0.001**
Nitrogen-dependent methane-oxidizing taxa	0.077	2.373	**0.031**	0.167	5.165	**0.001**	0.070	2.177	**0.042**	0.028	0.884	0.501	0.032	1.000	0.412

Bold values indicate statistical significance at *p*-value < 0.05. Euclidean distance index (chemical profile data) and Bray-Curtis distance index (microbial abundance data).

### Ammonia-oxidizing microbial community

**FIGURE 2:**
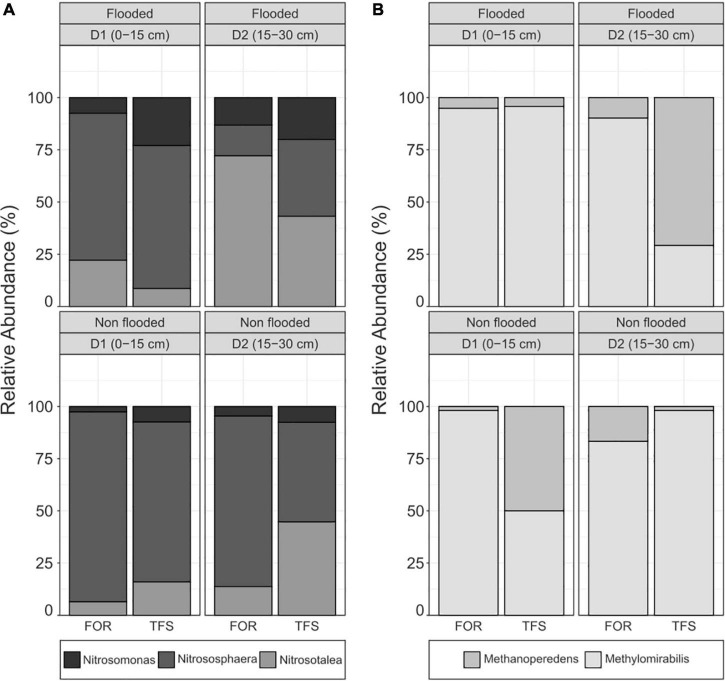
Relative abundance of **(A)** active ammonia-oxidizing genera and **(B)** active nitrogen-dependent anaerobic methane-oxidizing genera in each depth (0–15 and 15–30 cm), and season [Non-flood (NF) and Flood (F)], for contrasting land uses [Agroforest (TFS) and Forest (FOR)] in the Tocantins River plain.

Analysis of the soil layers [0–15 cm (D1) and 15–30 cm (D2)] in the FOR and TFS sites in the flood and non-flood seasons revealed the active presence of both ammonia-oxidizing archaea (AOA) and bacteria (AOB). The AOA community was composed of two distinct groups belonging to the archaeal phylum Thaumarchaeota, genus *Nitrososphaera* and genus *Nitrosotaleacea*, while the AOB community was composed of the members of the *Nitrosomonas* genus (Figure 2B). The archaeal and bacterial community composition is shown in the Supplementary Tables 1, 2.

The overall ammonia-oxidizing microbial community was mainly composed of AOA members, which presented a clear dominance over AOB in terms of absolute and relative abundances (Table 3 and Figure 2A). While highly dominant in every season, site, and depth, AOA was relatively more abundant during the non-flood season, where all sites had >90% of the ammonia-oxidizing community composed of AOA, with maximum relative abundance in D1 of the FOR site (97.46%). Unlike the AOA community, the AOB community displayed a higher relative abundance during the flood season, reaching up to 20% relative abundance at both depths of the TFS site, in contrast to the 7.36 and 7.58% abundance of AOB in the same site in the non-flood season for D1 and D2, respectively (Figure 2A).

**TABLE 3:** Absolute sequence abundance of active ammonia-oxidizing and anaerobic methane-oxidizing bacteria and archaea in each depth, site, and season in the Tocantins River plain based on 16S rRNA gene amplicon sequencing data.

Microbial group	Depth (cm)	Non-flood		Flood		Statistics			
				
		Forest (FOR)	Agroforest (TFS)	Forest (FOR)	Agroforest (TFS)	FOR vs. TFS		NF vs. F	
							
						NF	F	TFS	FOR
Ammonia-oxidizing taxa									
Total	0–15	1141.67a*a***a**	1245.00a*a***a**	871.33a*a***a**	597.33a*a***a**	ns	ns	ns	ns
	15–30	812.67a*a***a**	382.50a*a***a**	208.00a*a***a**	249.33a*a***a**				
Ammonia-oxidizing archaea (AOA)	0–15	1112.67a*a***a**	1153.33a*a***a**	806.67a*a***a**	460.00a*a***a**	ns	ns	ns	ns
	15–30	775.67a*a***a**	353.50a*a***a**	180.00a*a***a**	199.33a*a***a**				
Ammonia-oxidizing bacteria (AOB)	0–15	29.00a*b***a**	91.67a*a***a**	64.67a*a***a**	137.33a*a***a**	ns	ns	ns	ns
	15–30	37.00a*a***a**	29.00a*a***b**	28.00a*a***a**	50.00a*a***a**				
Nitrogen-dependent anaerobic methane-oxidizing taxa									
Total	0–15	226.33a*a***a**	1.33a*b***a**	430.00a*a***a**	285.67b*a***a**	ns	*	*	ns
	15–30	201.00a*a***a**	17.33a*b***a**	306.50a*a***a**	53.67a*b***a**				
*Candidatus* Methanoperedens	0–15	4.33a*a***a**	0.67a*a***a**	22.00a*a***a**	12.00a*a***a**	ns	*	ns	ns
	15–30	33.67a*a***b**	0.33a*b***a**	30.00a*a***a**	38.00a*a***a**				
*Candidatus* Methylomirabilis	0–15	222.00a*a***a**	0.67a*b***a**	408.00a*a***a**	273.67b*a***a**	ns	*	ns	ns
	15–30	167.33a*a***a**	17.00a*b***a**	276.50a*a***a**	15.67a*a***b**				

Values with different normal letters are significantly different (*p* < 0.05) based on Tukey’s HSD test. Tukey’s test was performed with F vs. NF for each land use and depth. Values with different italic letters were significantly different (*p* < 0.05) based on Tukey’s HSD test. Tukey’s test was performed with FOR vs. TFS for each season and depth. Values with different bold letters were significantly different (*p* < 0.05) based on Tukey’s HSD test. Tukey’s test was performed with D1 (0–15 cm) vs. D2 (15–30 cm) for each season and land use. *Significantly different (*p* < 0.05).

**FIGURE 3:**
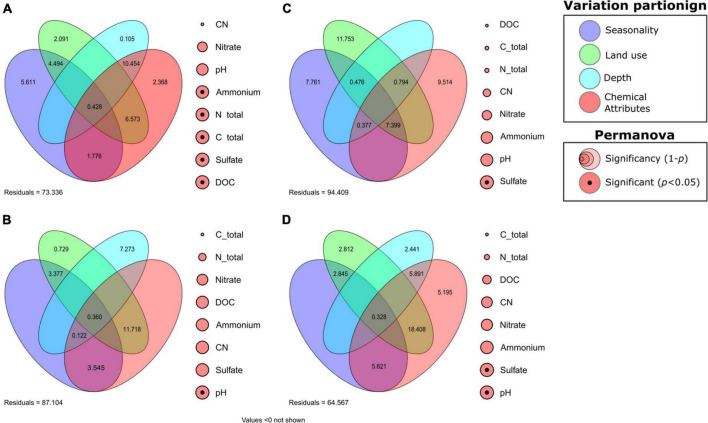
Variance partitioning of the active ammonia-oxidizing and nitrogen-dependent anaerobic methane-oxidizing communities, followed by the permutational analysis of variance of each chemical attribute. **(A)** Ammonia-oxidizing archaea (AOA), **(B)** ammonia-oxidizing bacteria (AOB), **(C)** *Methanoperedens*, and **(D)** *Methylomirabilis*.

Variance in AOB community composition was mainly affected by the interrelation between edaphoclimatic and soil chemical factors. The interaction between land use and soil chemical attributes had the highest influence on the variance (11.718%), whereas depth (7.273%), seasonality in combination with soil chemical attributes (3.545%), and seasonality in combination with land use (3.377%) also exhibited high contributions. The intersection between all four factors (0.360%) and between all three edaphic factors (0.122%) had the lowest values (Figure 3B). The majority of the AOB variance was considered residual, which corresponded to unaccounted factors (87.107%). Different variables were grouped to form the “soil chemical factors,” which contributed largely to AOB variance, and to better evaluate the impacts of these individual variables on the variance of AOB, a permutation analysis of variance (PERMANOVA) of each component of the soil chemical attributes was performed. The PERMANOVA analysis indicated that pH had the highest influence on the variance in AOB community composition within the pool of chemical variables, along with other factors (Figure 3B).

The soil chemical attributes alone accounted for little of the variance in the AOA community (2.368%), but their interaction with the other edaphic factors contributed to the majority of the AOA variance (21.599%). Depth in combination with soil chemical factors (10.454%), land use with soil chemical attributes (6.573%), seasonality (5.611%), and seasonality with land use (4.494%) had major contributions to AOA variance. In comparison, land use (2.091 %), seasonality in combination with soil chemical factors (1.776%), all four factors (0.428%), and depth (0.105%) had subtle effects on variance (Figure 3A). In contrast to AOB, the PERMANOVA analysis of the chemical attributes of AOA was significantly influenced by other soil chemical variables, such as dissolved organic carbon (DOC), total carbon, total nitrogen, sulfate, and ammonium (Figure 3A).

### Nitrogen-dependent anaerobic methane-oxidizing microbial community

We observed the presence of anaerobic methanotrophic archaea from the *Methanoperedens* genus and anaerobic methanotrophic bacteria from the *Methylomirabilis* genus in the soils from the floodplain of the Tocantins River across all seasons, land uses, and depths analyzed (Table 3 and Figure 2B). *Methylomirabilis* members were dominant across most samples, except for the D2 layer of the TFS site during the flood season, where *Methanoperedens* had a higher abundance (Figure 2B). Both methanotrophs had a higher absolute abundance during the flood season, especially in the D1 soil layer (Table 3).

The variance partition of the *Methanoperedens* members revealed that land use (11.753%), soil chemical factors (9.514%), and seasonality (7.761%) had strong influences on variance, as did the factors in combination (7.399%). The joint effect of land use with depth and soil chemical factors (0.794%), seasonality with land use and depth (0.476%), and seasonality with depth and soil chemical factors (0.377%) had small or negligible effects on variation (Figure 3C). PERMANOVA analysis was conducted to analyze the significance of each variable within the group of soil chemical attributes, and only sulfate was significant for *Methanoperedens* variation (Figure 3C).

For the variance partition of *Methylomirabilis* members, the joint effect of land use in combination with depth and soil chemical factors had the greatest influence on variation (18.408%). However, depth in combination with soil chemical factors (5.891%), soil chemical factors (5.195%), and seasonality in combination with soil chemical attributes (5.621 %) also contributed. Land use in combination with seasonality (2.845%), land use (2.812%), depth (2.441%), and the intersection of all four factors (0.328%) had a minor influence on the variation (Figure 3D). Finally, the PERMANOVA results revealed the significance of sulfate and pH.

### Interactions between ammonia oxidizers and nitrogen-dependent anaerobic methane oxidizers

**FIGURE 4:**
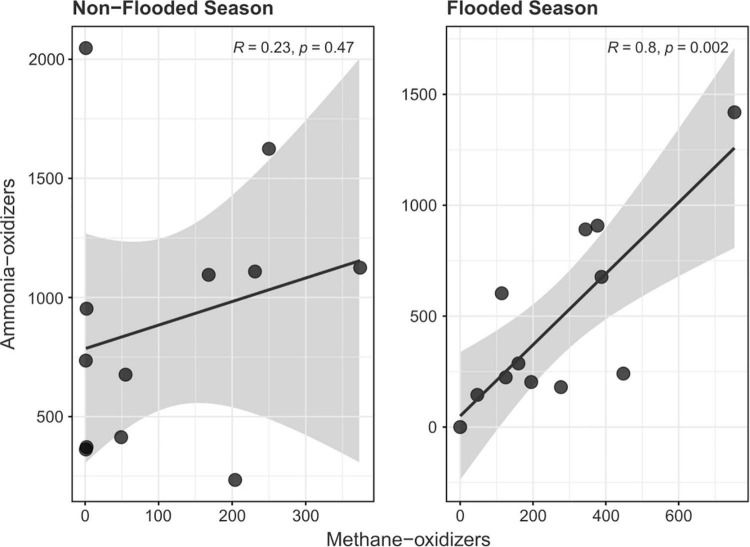
Correlation between active ammonia-oxidizing and nitrogen-dependent anaerobic ammonia-oxidizing communities during the flood and non-flood season.

The Pearson correlation between the ammonia-oxidizing and nitrogen-dependent anaerobic methane-oxidizing groups during the different seasons showed a positive and significant linear correlation in both groups only during the flood season (*R*^2^ = 0.80, *p* = 0.002). This positive correlation was accompanied by a decrease in the relative abundance of the bacterial phyla Actinobacteria, Planctomycetes, Firmicutes, Verrucomicrobia, and Bacteroidetes (Supplementary Table 3). A positive trend was also observed during the non-flood season, but it did not present a high correlation coefficient or significance at the 5% level (*R*^2^ = 0.23, *p* = 0.47) (Figure 4).

## Discussion

The results from this study reveal the importance of ammonia oxidizers in the production of oxidized nitrogen species for the N-DAMO community under the anoxic environment seasonally created by the flood of the Tocantins River. The importance of seasonal environmental changes is highlighted, as well as changes in depth and forest composition on the ammonia-oxidizing and N-DAMO community. Changes in both microbial communities were also notably tied to the soil chemical composition and to seasonal changes in the soil water saturation.

### Soil chemical profile

Cluster analysis of the chemical profiles of the Tocantins River floodplain soils showed significant dissimilarity in the chemical compositions in response to variations caused by seasonal changes, land use, and depth (Figure 1 and Table 2). Clear-water rivers, such as the Tocantins, drain areas with low erosion, are low in sediments and dissolved solids, and have intermediate fertility floodplains (Ríos-Villamizar et al., 2013). The low concentration of minerals in these rivers cannot provide significant fertility to the floodplain soils in the flood season; thus, most of the seasonal variations could relate to microbe-mediated processes that are enabled by flooding or by changes in the forest structure from FOR to TFS.

Under an anoxic environment created by increasing depth or flooding, nitrate (NO_3_^–^) is the preferred electron acceptor for the decomposition of organic matter, followed closely by nitrite (NO_2_^–^), manganese (IV), and iodate (IO_3_^–^) in terms of the expected energy yield (Froelich et al., 1979; Torres-Alvarado et al., 2005; Lam and Kuypers, 2011). Sulfate (SO_4_^2–^) can also be used as a terminal electron acceptor after all others have been removed, although it yields significantly less free energy (Δ*G*°) (Lam and Kuypers, 2011). Thus, NO_3_^–^ and SO_4_^2–^ concentrations decreased in the soil, with NO_3_^–^ being primarily consumed in both soil layers in the FOR and the D1 soil layer of the TFS site, whereas SO_4_^2–^ consumption was restricted to the D1 layer of the FOR and the D2 layer of the TFS site (Table 1). Both nitrate and sulfate reduction use H^+^ ions, which can be related to the increase in pH that occurs during the flood season, indicating the use of both ions as electron acceptors. Bento et al. (2021) also detected the presence of common nitrifiers, such as *Nitrososphaera* and *Nitrospira*, through amplicon sequencing analysis of the 16S rRNA gene, highlighting the importance of alternative electron acceptors for biogeochemical cycles during the flood season in the Amazonian floodplains. This chemical transformation can only occur in the absence of oxygen (O_2_), which can be achieved by an increase in depth and by flooding, which explains the high proportion of the variability explained by the changes in season and depth (Table 2).

Nitrification is a two-step process whereby ammonia (NH_3_^+^) is first oxidized to NO_3_^–^, which is then further oxidized to NO_2_^–^ (Prosser, 1990). In the first step of nitrification, ammonia oxidation is performed by the oxidation of NH_3_^+^ to hydroxylamine through the enzyme ammonia monooxygenase (AMO) (Hooper et al., 1997). O_2_ is also one of the requirements for the ammonia oxidation reaction, which can be limited by the lack of O_2_ caused by flooding. Thus, the accumulation of NH_3_^+^/ NH_4_^+^ during the flood season (Table 1) can also be explained by microbial activity, or in this case, the lack thereof.

### Ammonia-oxidizing and nitrogen-dependent anaerobic methane-oxidizing community

The ammonia-oxidizing community had a clear dominance of archaea over bacteria in all samples (Table 3 and Figure 2). This is expected in acidic soils, such as those found in the floodplains of the Tocantins River (Table 1), since most of cultivated AOB are highly sensitive to low pH environments (Zhang et al., 2012). The pH value was the major chemical attribute affecting AOB variance (Figure 3B), which is believed to be due to the low ammonia (NH_3_^+^) availability at low pH levels, as NH_3_^+^ is exponentially ionized to ammonium (NH_4_^+^), and NH_3_^+^ rather than NH_4_^+^ is thought to be the substrate for the ammonia monooxygenase enzyme, which catalyzes ammonia oxidation (Suzuki et al., 1974; Lehtovirta-Morley et al., 2011). Furthermore, kinetic studies on the isolated AOA *Nitrosopumilus maritimus* (strain SCM1) indicated a high ammonia affinity, suggesting that *Nitrosopumilus*-like AOA could not only out-compete AOB in ammonium-depleted environments, such as acidic soils, but could also effectively compete with other heterotrophs and phytoplankton for ammonia under these conditions (Martens-Habbena et al., 2009), The analysis of the membrane structure and genome of the acidophilic AOA isolate *Candidatus* Nitrosotalea devanaterra also indicated the presence of mechanisms for low pH tolerance, such as cation uptake, cytoplasmic buffering, and a cell membrane composition distinct from that of neutrophilic AOA (Lehtovirta-Morley et al., 2016). AOA members also encode a different type of N transporter, amt-NH_4_^+^ transporter, which differs from the Rh-NH_3_ transporters present in AOB, indicating better adaptation of AOA to acidic environments, such as those found throughout the Amazon floodplains (Offre et al., 2014; Lehtovirta-Morley et al., 2016).

Tropical soils are subjected to many environmental constraints, and the related microbes have a strong selective pressure to withstand and colonize these harsh and highly dynamic environments. Tropical soil bacterial communities contain more closely related taxa at the tip of the phylogenetic tree from more distantly related clades, suggesting a higher proportion of niche specialists (Bahram et al., 2018). Ammonia-oxidizing archaeal communities could behave in a similar way regarding the phylogenetic composition of the community, since, in acidic soils, ammonia oxidation is dominated by highly adapted archaea. These archaea are from closely related taxa of two evolutionarily distant acidophilic clades within the Thaumarchaeota phylum, one of which is related to the genus *Nitrosotalea* (Clusters 14 and 15) and the other to the genus *Nitrososphaera* (Cluster 11) (Gubry-Rangin et al., 2011, 2015; Macqueen and Gubry-Rangin, 2016). The evolution of these acidophilic clades appears to be driven mainly by pH, which is expected, as pH is one of the principal factors that determine niche differentiation between ammonia-oxidizing microorganisms (Gubry-Rangin et al., 2015).

Ammonia-oxidizing archaea are chemolithotrophs that can couple the oxidation of ammonia to C fixation, and the total nitrogen and ammonium/ammonia (hereafter referred to as “ammonia”) concentrations can directly influence this process (Stahl and de la Torre, 2012). AOA have a high affinity for ammonia and are well adapted to low nitrogen environments (Martens-Habbena et al., 2009). The AOA are autotrophs and they can fix carbon through a modified version of the 3- hydroxypropionate/4-hydroxybutyrate pathway (Walker et al., 2010; Könneke et al., 2014). AOB members of the Nitrosomonadaceae family are obligate autotrophs that can only fix carbon through the Calvin cycle (Clark et al., 2021). This could represent another evolutionary advantage for AOA in tropical floodplain soils, as DOC and total carbon had a significant effect on their variance (Figure 3A).

The nitrogen-dependent methane-oxidizing community had a higher abundance of *Methylomirabilis* than *Methanoperedens*, which can occur because of the absence of anammox taxa in our samples, which decreased the competition for NO_2_^–^ in the anoxic environment created by the flood, thus favoring other NO_2_^–^ reducers. NO_2_^–^ is especially important for *Methylomirabilis oxyfera*, as it couples NO_2_^–^ reduction to an intra-aerobic methane oxidation pathway (Ettwig et al., 2010), and has previously been identified in the floodplains of Amazonian rivers (Gabriel et al., 2020). Additionally, excessive competition can be the cause of the low abundance of *Methanoperedens*, as NO_3_^–^ is the preferred electron acceptor for anaerobic decomposition of organic material (Torres-Alvarado et al., 2005) and can also be used for nitrogen assimilation into the biomass of a multitude of organisms, including plants (Kuypers et al., 2018). In addition, NO_3_^–^ has high water solubility and can be easily lost in the soil profile through runoff (Robertson and Vitousek, 2009).

Members of both the Methanoperedenaceae and Methylomirabilaceae families have been previously co-enriched (Raghoebarsing et al., 2006), and in the absence of ammonia, which would inhibit anaerobic ammonia oxidation (anammox) activity and NO_2_^–^ consumption, the dominant microbial consortium in an anaerobic bioreactor fed with CH_4_ and NO_3_^–^ was that of the archaeon *Candidatus* Methanoperdens nitroreducens and the bacterium *Candidatus* Methylomirabilis oxyfera (Haroon et al., 2013), which were also detected in this floodplain (Gabriel et al., 2020; Bento et al., 2021; Gontijo et al., 2021).

The model of interaction between *Methanoperdens nitroreducens* and *Methylomirabilis oxyfera*, described by Haroon et al. (2013), may also be applied to the Tocantins River floodplain soils where high CH_4_ availability (Bento et al., 2021) and highly acidic (pH < 4) soils with low ammonia availability could inhibit anammox activity (Bai et al., 2015; Daverey et al., 2015) and allow for the cooperation between both methanotrophs. *Methanoperdens nitroreducens* can couple methane oxidation to reduce NO_3_^–^ to NO_2_^–^ (Raghoebarsing et al., 2006), which would fuel *Methylomirabilis oxyfera* as it couples methane oxidation to NO_2_^–^ reduction (Ettwig et al., 2009).

The AOM processes conducted by the members of both the *Methanoperedens* and *Methylomirabilis* genera can only occur under anaerobic conditions, and the total abundance of the nitrogen-dependent anaerobic methane oxidizers was considerably reduced during the non-flood season, approaching zero in D1 of the TFS site (Table 3). In contrast, the total abundance of ammonia oxidizers was reduced during the flood season due to O_2_ limitations caused by flooding but remained more abundant during the flood season than both nitrogen-dependent anaerobic methane oxidizers combined.

Ammonia-oxidizing archaea have been detected in oxygen-minimum zones (O_2_ ≤ 20 μM) and are considered providers of oxidized nitrogen species (nitrate and nitrite) to microbes in oxygen-minimum zones (Stahl and de la Torre, 2012). The supply of such nitrogen species can also fuel nitrogen-dependent anaerobic methane oxidation, especially in acidic environments such as the Tocantins River floodplain, where ammonium rather than ammonia is the most abundant ionic species, which could inhibit anammox activity. This inhibition would allow *Methanoperedens* and *Methylomirabilis* to undertake the process of nitrogen removal in the anaerobic and oxygen minimum zones, while AOA would remain active as they are better adapted to low pH and ammonium-rich environments and could present significantly higher oxygen affinities to withstand the flooded environment (Stahl and de la Torre, 2012). This interaction between both groups can only occur during the flood season, as biogeochemistry would not favor this combination in the non-flood season (Figure 4).

## Conclusion

The results of the current study showed that both ammonia oxidation and methanotrophy were deeply tied to the floodplain hydrological cycle, which is influenced by seasonal changes. In addition, both communities were influenced by forest composition and soil depth. Seasonal changes in soil water saturation were the main drivers of community composition, which in turn had significant effects on soil chemical factors. The ammonia-oxidizing community presented certain resilience to seasonal changes despite the relatively aerobic nature of the detected ammonia oxidizers and was also dominated by AOA members, as expected for acidic soils. The nitrogen-dependent anaerobic CH_4_-oxidizing community presented a clear difference between seasons, with greater abundance during the flood season, and was affected by changes in forest composition and soil depth. Based on 16S rRNA transcript amplicon sequencing data and statistical analysis, aerobic ammonia oxidizers were significantly related to the nitrogen-dependent anaerobic CH_4_-oxidizing community during the flood season, as ammonia oxidation can provide oxidized nitrogen members that can be coupled to anaerobic CH_4_ oxidation. We suggest that these theoretical relationships can be validated in future studies to provide insight into the complexity of the carbon and nitrogen cycles in the Amazonian floodplain and the carbon and nitrogen cycling microbial community responses to environmental changes, which can include those related to ongoing climate change.

## Data availability statement

The datasets presented in this study can be found in online repositories. The name of the repository and accession number can be found below: National Center for Biotechnology Information (NCBI) BioProject, https://www.ncbi.nlm.nih.gov/bioproject/, PRJNA732086.

## Author contributions

AN, PB, and VN contributed to the conception and design of the study. AN, GM, and DB organized the database. GM, GV, and TV performed the statistical analysis. GM, AN, and GG wrote the first draft of the manuscript. AV, LO, VN, PB, CM, and ST wrote sections of the manuscript. All authors contributed to manuscript revision, read, and approved the submitted version.

## Footnotes

1
https://support.illumina.com

2
https://www.arb-silva.de
