# Supplementary material for: Molecular evidence for stimulation of methane oxidation in Amazonian floodplains by ammonia-oxidizing communities
Source: Front Microbiol. 2022 Aug 1;13:913453. doi: 10.3389/fmicb.2022.913453 (PMC9376453; doi:10.3389/fmicb.2022.913453)
Supplement: Supplementary file 1 [file Data_Sheet_1.PDF]

Supplementary Material

1 Supplementary Figures and Tables

1.1 Supplementary Figures

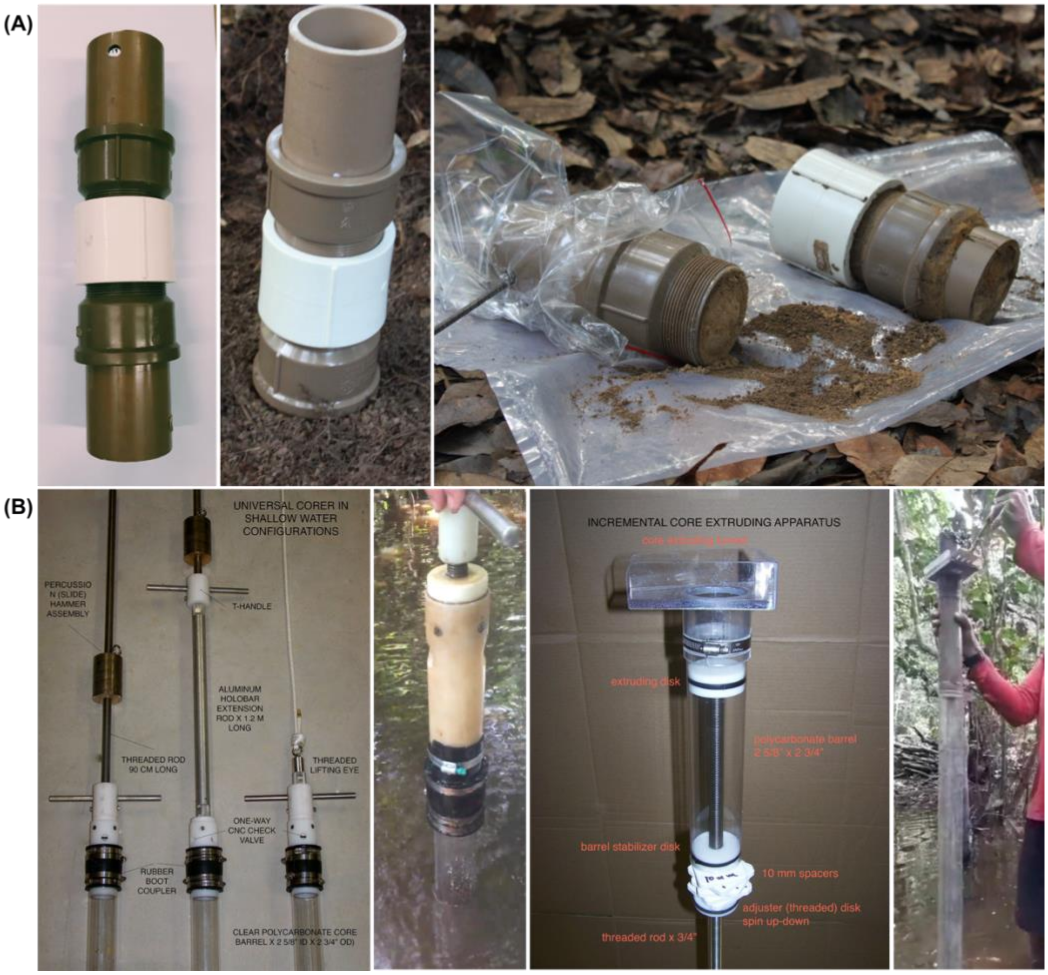

Supplementary Figure 1. Soil core samplers used to collect undisturbed soil core samples for physicochemical and microbiological investigations at the drying (A – aseptic cylindrical core 0-30 cm soil layer and 10 cm diameter with 0-15 cm fractionable core) and flooding (B – universal hand core sediment sampler with 0-15 cm adjustable core cutter) regimes of the floodplains

1.2 Supplementary Tables

Supplementary Table 1. Relative sequence abundance of active archaeal groups based on 16S rRNA gene amplicon sequencing data (2,500 sequences) for each of the three libraries per sampling point in each of the two seasonal sampling periods classified by using the QIIME (Quantitative Insights Into Microbial Ecology) software based on the SILVA database and sorted with >97% similarity into operational taxonomic units (OTUs) using closed reference OTU picking approach

Taxonomical group	Depth (cm)	Flooded		Non-flooded		Statistics			
		Forest (FOR)	Agroforest (TFS)	Forest (FOR)	Agroforest (TFS)	FOR vs. TFS		NF vs. F	
						NF	F	TFS	FOR
Crenarchaeota	0-15	751[1] ± 276,3[2] a*a***a**	645,3 ± 276,5a*a***a**	753 ± 345,5a*a***a**	380,7 ± 363,5a*a***b**	ns	ns	ns	ns
	15-30	547 ± 128a*a***a**	69,7 ± 120,7b*b***b**	702,7 ± 131,7a*b***a**	1296,5 ± 103,5a*a***a**				
Euryarchaeota	0-15	456 ± 275,8a*a***a**	233 ± 67,5a*a***a**	206,3 ± 42,2a*a***a**	199,7 ± 160,1a*a***a**	ns	ns	ns	ns
	15-30	1400,5 ± 143,5a*a***b**	458,7 ± 794,4a*a***a**	679,7 ± 676,4a*a***a**	279,5 ± 0,5a*a***a**				
Thaumarchaeota	0-15	883 ± 482,7a*a***a**	490 ± 263,5a*a***a**	1165,7 ± 435,8a*a***a**	1303,7 ± 584,7a*a***a**	ns	ns	ns	ns
	15-30	450 ± 3a*a***a**	273,3 ± 473,4a*a***a**	920,7 ± 453,5a*a***a**	595,5 ± 27,5a*a***a**				
Nanoarchaeota	0-15	23 ± 27,2a*a***a**	58,3 ± 35,9a*a***a**	0,3 ± 0,6a*a***a**	1 ± 1,7a*a***a**	ns	ns	ns	ns
	15-30	7 ± 7a*a***a**	0 ± 0a*a***a**	1,7 ± 2,1a*a***a**	0 ± 0a*a***a**				
Asgardeota	0-15	0,7 ± 1,2a*a***a**	11 ± 19,1a*a***a**	4 ± 5,3a*a***a**	0 ± 0a*a***a**	ns	ns	ns	ns
	15-30	0 ± 0a*a***a**	0 ± 0a*a***a**	4 ± 6,1a*a***a**	0,5 ± 0,5a*a***a**				
Diapherotrites	0-15	1,7 ± 2,9a*a***a**	3 ± 5,2a*a***a**	11 ± 19,1a*a***a**	0,3 ± 0,6a*a***a**	ns	ns	ns	ns
	15-30	0 ± 0a*a***a**	0 ± 0a*a***a**	2,7 ± 2,3a*a***a**	0 ± 0a*a***a**				
Hadesarchaeota	0-15	0,7 ± 1,2a*a***a**	0 ± 0a*a***a**	0 ± 0a*a***a**	0 ± 0a*a***a**	ns	ns	ns	ns
	15-30	0 ± 0a*a***a**	0 ± 0a*a***a**	0 ± 0a*a***a**	0 ± 0a*a***a**				
Hydrothermarchaeota	0-15	0 ± 0a*a***a**	0 ± 0a*a***a**	0 ± 0a*a***a**	0 ± 0a*a***a**	ns	ns	ns	ns
	15-30	0 ± 0a*a***a**	0 ± 0a*a***a**	2,7 ± 4,6a*a***a**	0,5 ± 0,5a*a***a**				

FOR, Forest; TFS, Traditional Farming System.

[1]Average for each of three replicates soil.

[2]Standard deviation of the average for each of three replicates soil.

Values with the same letters were not significantly different ($p<0.05$) based on upon a Tukey's HSD test.

Supplementary Table 2. Relative sequence abundance of active bacterial groups based on 16S rRNA gene amplicon sequencing data (20,000 sequences) for each of the three libraries per sampling point in each of the two seasonal sampling periods classified by using the QIIME (Quantitative Insights Into Microbial Ecology) software based on the SILVA database and sorted with >97% similarity into operational taxonomic units (OTUs) using closed reference OTU picking approach

Taxonomical group	Depth (cm)	Flooded		Non-flooded		Statistics			
		Forest (FOR)	Agroforest (TFS)	Forest (FOR)	Agroforest (TFS)	FOR vs. TFS		NF vs. F	
						NF	F	TFS	FOR
Proteobacteria (total)	0-15	5710,7[(1)] ± 2330,4[(2)] aaa	6335,7 ± 1972,7aaa	6451 ± 1054aaa	5800,3 ± 2265,1aaa	ns	ns	ns	ns
	15-30	2871 ± 859aba	5198,5 ± 294,5aaa	5087,3 ± 2027,7aaa	5816,3 ± 1957,4aaa				
Alphaproteobacteria	0-15	2428,3 ± 731,4aaa	2337,7 ± 261,4aaa	2606,7 ± 972,6aaa	3157,7 ± 1522,7aaa	ns	ns	ns	ns
	15-30	552,5 ± 403,5aab	1189 ± 638aab	1714 ± 830,3aaa	2043 ± 280,6aaa				
Deltaproteobacteria	0-15	2098,7 ± 1110,5aaa	2811,7 ± 1762,4aaa	1323 ± 907,4aaa	891 ± 277,9aaa	ns	ns	ns	ns
	15-30	1142,5 ± 607,5aaa	386 ± 382aaa	1004 ± 340aaa	757,7 ± 382,4aaa				
Gammaproteobacteria	0-15	1183,7 ± 520,3aaa	1186,3 ± 393,3aab	2521,3 ± 1542,8aaa	1751,7 ± 636,3aaa	ns	ns	ns	*
	15-30	1176 ± 152aba	3623,5 ± 725,5aaa	2369,3 ± 1052,5aaa	3015,7 ± 1306,3aaa				
Acidobacteria	0-15	4732,7 ± 1307,2aaa	4550,7 ± 357,6aaa	2398,3 ± 1361,3aaa	2213,3 ± 934,5baa	ns	ns	ns	ns
	15-30	2858,5 ± 1633,5aaa	1855,5 ± 1076,5aab	3339,7 ± 346,5aaa	1639 ± 605,8aba				
Actinobacteria	0-15	1502,3 ± 1931,2aaa	1178,3 ± 1349,6aaa	845 ± 292,7aaa	1641,7 ± 815,8aaa	ns	ns	ns	ns
	15-30	675,5 ± 82,5baa	1421,5 ± 1244,5aaa	1097,3 ± 239,4aaa	1303,7 ± 232,4aaa				
Planctomycetes	0-15	1078 ± 134,4aaa	1763,7 ± 875,8aaa	2651,3 ± 1547,3aaa	3352,7 ± 557,2aaa	ns	ns	ns	*
	15-30	345,5 ± 332,5bab	1117 ± 1117aaa	2364,7 ± 994,4aaa	1639 ± 553,3aab				
Chloroflexi	0-15	2116,7 ± 1332,8aa	1867,7 ± 1016aaa	1310,3 ± 634,7aaa	1488,7 ± 552,5aaa	ns	ns	ns	ns
	15-30	2239 ± 138aaa	953,5 ± 559,5aba	2849,7 ± 828,5aaa	2595,7 ± 1300,8aaa				
Firmicutes	0-15	1164,3 ± 458,2aaa	862,7 ± 107,8aaa	3339,7 ± 5161,5aaa	730,3 ± 1020,8aaa	ns	ns	ns	ns
	15-30	2758 ± 1979aaa	6412 ± 4243aaa	210 ± 182,7aba	720 ± 243,6aaa				
Verrucomicrobia	0-15	425 ± 218,7aaa	1046 ± 333,9aaa	537 ± 180,2aaa	1458,3 ± 564,3aaa	ns	ns	ns	ns
	15-30	372,5 ± 359,5aaa	486,5 ± 484,5aaa	368,7 ± 271,8aaa	411,3 ± 353,1aaa				
Bacteroidetes	0-15	389 ± 273,1aaa	712,7 ± 437,2aaa	259 ± 229,6aaa	222 ± 174,9aaa	ns	*	ns	ns
	15-30	252 ± 144aaa	997,5 ± 256,5aaa	178,3 ± 59,2aaa	513,7 ± 716,5aaa				
Nitrospinae	0-15	163,7 ± 92,6aaa	138,3 ± 70,9aaa	131 ± 108,3aaa	63,7 ± 40,5aaa	*	ns	ns	ns
	15-30	82 ± 38baa	76,5 ± 71,5aaa	246,3 ± 76,2aaa	71 ± 107,5aaa				
Cyanobacteria	0-15	186,7 ± 154aaa	166,3 ± 186,2aaa	157 ± 123aab	317,3 ± 251,6aaa	ns	ns	ns	ns
	15-30	136 ± 1baa	18,5 ± 18,5aba	680,3 ± 290,2aaa	1390 ± 1159,2aaa				
Rokubacteria	0-15	420,7 ± 309,6aaa	316 ± 109aaa	226,7 ± 164aaa	493,7 ± 441aaa	ns	*	ns	ns
	15-30	495 ± 117aaa	66 ± 17bbb	297 ± 102,5aaa	450 ± 170,6aaa				
Others	0-15	1100,3 ± 383,6aaa	652,7 ± 47,8baa	407,3 ± 207,6aba	1099,3 ± 189,4aab	ns	ns	*	ns
	15-30	891,5 ± 541,5aaa	734,5 ± 23,5baa	1197,3 ± 1085,8aaa	1944,3 ± 323,6aaa				

FOR, Forest; TFS, Traditional Farming System.

(1)Average for each of three replicates soil.

(2)Standard deviation of the average for each of three replicates soil.

Values with the same letters were not significantly different ($p<0.05$) based on upon a Tukey's HSD test.

Supplementary Table 3. Pearson's correlation coefficients between abundance of ammonia- and methane-oxidizers and archaeal and bacterial taxonomic groups

Prokaryotic community	Non-flooded season		Flooded season	
	**Ammonia-oxidizers**	**Methane-oxidizers**	**Ammonia-oxidizers**	**Methane-oxidizers**
**Archaeal groups**				
Crenarchaeota	-0.76848	0.06866	0.39436	0.65806
Euryarchaeota	-0.63079	0.30745	0.13841	0.47601
Thaumarchaeota	0.99189	0.1045	0.83919	0.83266
Nanoarchaeota	0.22244	0.2484	0.4482	0.46872
Asgardeota	-0.00656	0.32345	0.24098	0.18683
Diapherotrites	0.01065	0.07844	0.4454	0.18601
Hadesarchaeota	0	0	0.30944	0.14817
Hydrothermarchaeota	-0.61318	0.23312	0	0
**Bacterial groups**				
Proteobacteria (total)	0.43322	-0.05403	0.36699	0.01947
Alphaproteobacteria	0.81051	-0.22893	0.29202	0.08071
Deltaproteobacteria	0.46494	0.51296	0.50319	0.66758
Gammaproteobacteria	-0.42059	-0.121	-0.40064	-0.6193
Acidobacteria	0.032	0.55631	0.33007	0.5976
Actinobacteria	-0.28835	-0.29581	-0.60525	-0.4602
Planctomycetes	0.60247	0.11092	-0.21975	-0.21912
Chloroflexi	-0.48772	0.22555	-0.28107	0.1858
Firmicutes	-0.03056	-0.21912	-0.39607	-0.49519
Verrucomicrobia	0.45935	-0.46071	-0.11247	-0.00416
Bacteroidetes	-0.3292	-0.32719	-0.21705	-0.55218
Nitrospinae	0.14113	0.76842	0.21521	0.44132
Cyanobacteria	-0.32407	-0.04096	0.45525	0.66195
Rokubacteria	-0.47764	-0.20296	0.41662	0.88304
Others	-0.64368	-0.29778	-0.24272	0.09457
